# Herpes simplex virus type 2 and HIV disease progression: a systematic review of observational studies

**DOI:** 10.1186/1471-2334-13-502

**Published:** 2013-10-28

**Authors:** Darrell Hoi-San Tan, Kellie Murphy, Prakesh Shah, Sharon Lynn Walmsley

**Affiliations:** 1 Division of Infectious Diseases, St. Michael’s Hospital, 30 Bond St. 4CC – Room 4-179, Toronto, ON M5B 1W8, Canada; 2 Division of Infectious Diseases, University Health Network, 585 University Ave. 13-N, Toronto, ON M5G 2N2, Canada; 3 Division of Infectious Diseases, University of Toronto, 200 Elizabeth St, Toronto, ON M5G 2C4, Canada; 4 Institute of Health Policy, Management and Evaluation, University of Toronto, 155 College St., Suite 425, Toronto, ON M5T 3M6, Canada; 5 Department of Maternal Fetal Medicine, Mount Sinai Hospital, 600 University Avenue, Toronto, ON M5G 1X5, Canada; 6 Department of Pediatrics, University of Toronto, 600 University Ave., Room 775-A, Toronto, ON M5G 1X5, Canada

**Keywords:** Herpes simplex virus, Human immunodeficiency virus, Disease progression, Viral load, CD4 count, Seropositivity, Systematic review

## Abstract

**Background:**

Herpes simplex virus type 2 (HSV-2) is a common co-infection among HIV-infected adults that is hypothesized to accelerate HIV disease progression.

**Methods:**

We searched Medline, EMBASE, relevant conference proceedings (2006–12) and bibliographies of identified studies without language restriction for cohort studies examining the impact of HSV-2 on highly active antiretroviral therapy-untreated HIV disease in adults. The exposure of interest was HSV-2 seropositivity or clinical/laboratory markers of HSV-2 activity. The primary outcome was HIV disease progression, defined as antiretroviral initiation, development of AIDS/opportunistic infection, or progression to CD4 count thresholds (≤200 or ≤350 cells/mm^3^). Secondary outcomes included HIV plasma viral load and CD4 count.

**Results:**

Seven studies were included. No definitive relationship was observed between HSV-2 seropositivity and time to antiretroviral initiation (n=2 studies), CD4≤350 (n=1), CD4≤200 (n=1), death (n=1), viral load (n=6) or CD4 count (n=3). Although two studies each observed trends towards accelerated progression to clinical AIDS/opportunistic infection in HSV-2 seropositives, with pooled unadjusted hazard ratio=1.85 (95% CI=1.12,3.06; I^2^=2%), most OIs observed in the study for which data were available can occur at high CD4 counts and may not represent HIV progression. In contrast, a single study HSV-2 disease activity found that the presence of genital HSV-2 DNA was associated with a 0.4 log copies/mL increase in HIV viral load.

**Conclusions:**

Despite an observation that HSV-2 activity is associated with increased HIV viral load, definitive evidence linking HSV-2 seropositivity to accelerated HIV disease progression is lacking. The attenuating effects of acyclovir on HIV disease progression observed in recent trials may result both from direct anti-HIV activity as well as from indirect benefits of HSV-2 suppression.

## Background

Herpes simplex virus type 2 (HSV-2) is a common pathogen that co-infects over half of HIV-infected adults [1], and may accelerate HIV disease by increasing plasma HIV viral load [2,3]. Some authors therefore suggest that HIV-infected patients be routinely screened for HSV-2 antibodies, and that co-infected individuals be offered chronic suppressive HSV therapy [4]. Indeed, two recent clinical trials among HIV, HSV-2 co-infected adults in Sub-Saharan Africa have demonstrated a modest attenuation in HIV disease progression with the anti-HSV medication acyclovir 400mg twice daily [5,6]. Two potential mechanisms for this finding have been advanced. The leading hypothesis is that by suppressing HSV-2 activity, acyclovir reverses the adverse impact of HSV-2 co-infection on HIV disease progression. More recently, however, in vitro data have suggested that acyclovir may have direct anti-HIV activity [7,8], raising the possibility that anti-HIV effects may have also contributed to the trial findings.

To further investigate these issues, and to inform the counseling of HIV patients regarding the impact of co-infection on their HIV disease, we undertook a systematic review of observational cohort studies addressing the impact of HSV-2 on longitudinal measures of HIV disease progression. The primary objective was to summarize the impact of HSV-2 co-infection on antiretroviral therapy (ART)-untreated HIV disease progression among HIV-1-infected adults. Secondary objectives were to summarize the relationship between HSV-2 co-infection (including both seropositivity and measures of HSV-2 disease activity) and HIV plasma viral load and CD4 count.

## Methods

Methods of the analysis and inclusion criteria were specified in advance and documented in a protocol available from the authors.

### Study and participant criteria

Cohort studies examining the impact of HSV-2 co-infection on progression of ART-untreated HIV disease over ≥6 months, HIV plasma viral load, or CD4 count were considered for inclusion. Studies must have included HIV-1-infected adults not using ART (mono- or combination therapy). However, a single study including short-course zidovudine for the prevention of mother-to-child HIV transmission (week 34–36 of pregnancy until delivery) was included, because this short duration of exposure was unlikely to impact on disease progression over the 18 month median duration of follow-up. Participants must not have been using chronic suppressive anti-HSV therapy (acyclovir, valacyclovir and/or famciclovir) or other agents with anti-HSV activity (eg. ganciclovir, valganciclovir, cidofovir, foscarnet).

### Exposure

The exposure of interest was HSV-2 co-infection. Because HSV-2 is incurable and frequently asymptomatic, this exposure can only be accurately ascertained through HSV type-specific serologic assays that detect antibody to glycoprotein G. Studies using less accurate serologic assays such as those based on whole virus or crude antigen preparations were excluded.

Alternative laboratory measures of HSV-2 disease activity with good specificity were also examined, including viral culture and viral shedding detected by polymerase chain reaction (PCR), but studies using clinical history only were excluded because such anamnestic methods are of limited sensitivity.

### Outcome measures

The primary outcome of interest was HIV disease progression. Because multiple valid measures of this concept exist and have changed over time, it was important to include different definitions of this outcome and consider each one separately, including a) ART initiation b) development of AIDS or first opportunistic infection (OI), c) progression to CD4 count ≤200 cells/mm^3^, and d) progression to CD4 count ≤350 cells/mm^3^. Secondary outcomes were HIV plasma viral load and CD4 count since these are critical laboratory markers for assessing HIV disease progression. Since viral loads fluctuate around a stable ‘set-point’ in individual patients, the main viral load measures of interest were time-averaged viral loads collected over ≥6 months, or changes in viral load at author-defined time points. The main CD4 count measure of interest was the rate of change per unit time. Within-patient changes in viral load and CD4 count associated with HSV-2 disease activity over time were also collected.

### Search methods for identifying studies

Eligible publications were identified through electronic searches of Medline (January 1950-April week 3, 2013) and EMBASE (1980–2013 week 16) followed by removal of duplicate citations. Details of the Medline search strategy are provided in Additional file 1. Manual searches of the following conference proceedings from 2006–2012 were also conducted: Conference on Retroviruses and Opportunistic Infections, International AIDS Society Conference on HIV Pathogenesis, Treatment and Prevention; IAS World AIDS Conference, Interscience Conference on Antimicrobial Agents and Chemotherapy, Infectious Diseases Society of America Annual Meeting, and the International Society for Sexually Transmitted Disease Research. Reference lists of identified articles were also used. No language restrictions were applied. We did not search grey literature or dissertation indices.

### Selection of studies and data extraction

All publications and abstracts that appeared to meet eligibility criteria were retrieved. Two authors (DHST, KM) independently assessed studies for inclusion in an unblinded standardized manner, and independently extracted data onto standardized forms, with disagreements resolved by consensus and involvement of other authors. Information was extracted from each included study on: 1) characteristics of study participants, including geographic location, sex, baseline CD4 count, baseline HIV viral load, 2) methods of HSV-2 ascertainment, including details of laboratory assays as appropriate, and 3) outcome measures, including time-to-event data, CD4 counts, and viral load, along with the follow-up time for the study. Attempts to contact authors to supply missing data were made on up to three occasions; if information was not available, data were assumed to be missing at random.

### Assessment of risk of bias

Assessment of risk of bias was performed for each study to explain heterogeneity in results and perform sensitivity analyses. A list of important study features that may impact on bias was developed by reviewing the STROBE statement and other published criteria [9,10]. This approach was used because a previously published systematic review of tools for assessing the quality and susceptibility to bias in observational studies was unable to identify a single obvious candidate tool for this purpose, and similarly references the STROBE statement as a valuable starting point for quality assessment [11]. The risk of bias for each study was assessed systematically using the criteria in Table 1. Assessments were not blinded to articles for feasibility reasons.

**Table 1:** **Criteria for assessing the risk of bias in included studies**^**a**^

**Criterion**	**Risk of bias**		
	**Low**	**Moderate**	**High**
**Eligibility**	· Recruited from general HIV-infected population, eg. HIV clinics	· Recruited from moderately selected population, eg. STI clinics	· Highly selected population (eg. FSW, active GUD)
			· Unclear selection criteria applied
**HSV-2 ascertainment**	· High quality type-specific serology assay	· Culture-based diagnosis	· Methods unclear
	· PCR assay	· Clinical diagnosis	
**Endpoint ascertainment**	· Similar between groups	· Similar between groups	· Methods unclear
	· Regular timing	· Irregular timing	
**Confounding**	· Accounted for ART, and	· Some of these confounders accounted for	· None of these confounders accounted for or unclear
	· Accounted for acyclovir, and		
	· Accounted for CD4/stage of HIV disease		
**Analysis**	· Sample size or power calculation done, and	· No concerns with analysis	· Problems identified with analysis
	· No concerns with analysis		
**Attrition**	· Minimal attrition (<10%) and	· Moderate attrition (10-20%)	· High attrition (>20%)
	· Attrition explained		· Attrition not explained
**OVERALL**	· Most items at low risk of bias, including both HSV-2 ascertainment and confounding	· Most items at low to moderate risk of bias	· Most items at moderate risk of bias
	· Not more than two items at moderate risk of bias	· No item at high risk of bias	· At least one item at high risk of bias

^a^FSW = female sex workers; GUD = genital ulcer disease; STI = sexually transmitted infection.

### Analysis

The primary expected summary measure relating HIV disease progression to HSV-2 co-infection was the hazard ratio. Where appropriate, hazard ratios were pooled in meta-analyses using random effects models, with weighting of studies according to the DerSimonian-Laird method. Cochran's Q test was used to test for heterogeneity between studies at the 0.10 level of significance. The I-squared statistic was used to quantify the degree of heterogeneity. For viral load and CD4 count outcomes, meta-analysis was planned using the weighted mean difference, but available data were inappropriate for analysis due to differences in study methodology and reporting. Formal tests to assess for the possibility of publication bias were not performed given the variety of ways in which outcomes were reported. All statistical analyses were performed using Review Manager version 5. Data were synthesized descriptively when not appropriate for meta-analysis.

Subgroup analyses were planned to examine incident rather than prevalent HSV-2 infection, incident versus prevalent HIV infection, geographic region, HSV-1 co-infection, and use of any antiretrovirals. Sensitivity analyses were planned in which the results of studies would be considered in groups, according to the risk of bias.

## Results

### Studies included in the review

**Figure 1:**
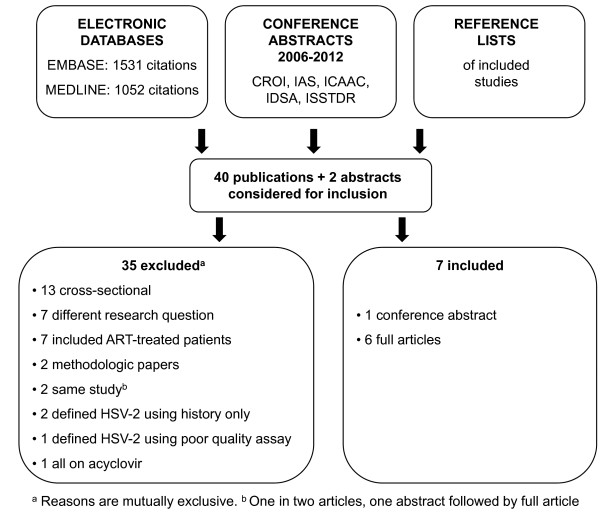
Flow chart for identifying studies.

The electronic search strategy identified 1052 articles through MEDLINE and 1531 through EMBASE (Figure 1). Of these, 38 appeared to meet eligibility criteria. Two potentially eligible articles were identified from reference lists, and two more from conference proceedings. Of the resulting 42 studies, 7 were ultimately included (Table 2) [12-18]. Thirty-five papers were excluded because they were cross-sectional in design (n=13), addressed a different research question (n=7), included ART-treated patients (n=7) were methodologic in nature (n=2), were duplicate publications (n=2), used clinical history (n=2) or a poor quality serologic assay (n=1) to define HSV-2 status, or included only patients receiving acyclovir (n=1).

**Table 2:** **Characteristics of included studies and assessment of the risk of bias**^**a**^

**Study**	**Study Characteristics**						**Assessment of the risk of bias**							
	**Population**	**N**^**b**^	**% Male**	**Baseline CD4**	**Baseline VL**	**Follow-up time**	**Eligibility**	**HSV-2 Ascertain-ment**	**Endpoints**	**Confoun-ding**	**Analysis**	**Attrition**	**OVERALL**	**Impact of bias**^**c**^
Barnabas 2011	Men, International	88	100	NR	NR	NR	Moderate	Low	Low	Moderate	Moderate	Moderate	Moderate	↓
Cachay 2007	Male HIV seroconverters, USA	294	100	497	4.97	205 days	Low	Low	Low	Low	Moderate	Low	Low	N/A
Cachay 2008	Male HIV seroconverters, USA	119	100	520	5.04	779 days	Low	Low	Moderate	Low	Moderate	Low	Low	N/A
Crum-Cianflone 2006	USA	367	92	499	NR	NR	Low	Moderate	Moderate	High	Moderate	Moderate	High	?
Nagot 2008	Women, Burkina Faso	140	0	443	4.48	12 weeks	Moderate	Low	Low	Low	Moderate	Low	Low	N/A
Roxby 2011	Pregnant women, Kenya	296	0	422	4.75	18 months	Moderate	Low	Low	Moderate	Moderate	Moderate	Moderate	↓
Suligoi 2001	HIV sero-converters, Italy	380	78	NR	NR	7.8 years	Low	Low	Moderate	High	Moderate	High	High	?

^a^Median values are reported unless noted otherwise. When values were not reported for overall population, a weighted average was taken.

^b^N reported for patients included in relevant comparisons only.

^c^Assessed only for studies deemed at moderate or high risk of bias. N/A = not applicable (ie. study at low risk of bias), ↓ indicates bias towards null, ? indicates uncertain bias of direction.

### Assessment of risk of bias

The quality of included studies was variable (Table 2). Eligibility criteria were generally appropriate and well described, with most included studies conducted among unselected cohorts of HIV-infected adults. All studies employing serology to ascertain HSV-2 status used high quality assays, although the timing of HSV-2 testing relative to the period of follow-up was unclear in one report [15]. The single study employing a marker of HSV-2 disease activity in the analysis used HSV-2 polymerase chain rection (PCR) to detect the presence of reactivating virus in a cohort of HSV-2 seropositive participants [16]. Outcome assessments were general well performed, although their timing was irregular or unclear in some reports [14,15]. Several studies did not report on or adequately adjust for relevant confounders including anti-HSV medications [12,15,17,18], and baseline CD4 count [15]. No study included a formal sample size calculation, and attrition was inconsistently described, but analytic techniques were generally appropriate.

For studies at moderate or high risk of bias, a qualitative assessment was made regarding the likely direction of bias(es). For instance, studies failing to systematically account for anti-HSV medications may be biased towards the null [12,15,17,18].

### Studies evaluating HSV-2 seropositivity

#### Disease progression outcomes

Four studies evaluated the relationship between HSV-2 seropositivity and HIV disease progression outcomes (Table 3). Two considered ART initiation as an endpoint, and neither observed an association with HSV-2 seropositivity; these studies were at moderate [12] and high [15] risk of bias respectively but the likely direction of this bias was difficult to ascertain. In addition, different definitions of ART were used, as shown. Data were therefore not appropriate for meta-analysis [12,15].

**Table 3:** **Impact of HSV-2 seropositivity on HIV disease progression**^**a**^

**Study**	**Population**	**N**	**HSV-2 Ascertainment**	**Results**				
				**Outcome**	**HSV2 positive**	**HSV2 negative**	**Difference**	**Effect**^**b**^
Barnabas 2011	HIV seroconverters, Americas	87	Serology: Western Blot	Initiation of HAART	NR	NR	aHR = 1.3^c,^ (95% CI = 0.5, 3.2)	↔
Crum-Cianflone 2006	USA	367	Serology: Focus	Time from HIV diagnosis to initiation of any ART^d^	18 months	9 months	p = 0.10	↔
					(range **=** 1,156)	(range = 1,96)		
		271		Proportion with CD4≤350 or 18% at 1 year of follow-up^e^	62/144 = 43%	66/127 = 52%	OR = 0.70, p = 0.15	↔
		221		Proportion with CD4≤350 or 18% at 3 years of follow-up^e^	84/118 = 71%	75/103 = 73%	OR = 0.92, p = 0.88	↔
Roxby 2011	Postpartum women, Kenya	296	Serology: Focus	CD4≤200	50/254 = 20%	7/42 = 17%	aHR = 1.16^f,^ (95% CI = 0.52,2.56), p = 0.72	↔
				First OI	43/254 = 17%	2/42 = 5%	aHR = 3.83^f^, (95% CI = 0.93,15.83), p = 0.06	Faster?
				Death	17/254 = 7%	2/42 = 5%	aHR = 1.33^f,^ (95% CI = 0.32,6.05), p = 0.66	↔
Suligoi 2001	Italy	380	Serology: Pockit	AIDS	NR	NR	HR = 1.68, (95% CI = 1.19,2.37), Adjusted^g^ HR = 1.13, (95% CI = 0.76, 1.70)	Faster?

^a^CI = confidence interval, HAART = highly active antiretroviral therapy, HR = hazard ratio, NR = not reported, OI = Opportunistic infection, OR = odds ratio.

^b^Arrows show directions of association with HSV-2; ‘?’ denotes borderline statistical significance or clinically significant differences not meeting statistical significance.

^c^Adjusted for adenovirus 5 immunity, region, circumcision status, age, race, HLA group, trial intervention assignment (vaccine / placebo).

^d^Includes any ART (monotherapy, non-HAART combination therapy and HAART).

^e^Defined as CD4 count <350 cells/mm^3^ or 18%.

^f^Adjusted for baseline CD4 count.

^g^Adjusted for age at HIV seroconversion, exposure category, sex.

The latter of these studies also reported on the proportion of individuals reaching a CD4 count ≤350 cells/mm^3^ or 18% at various time points and found no difference according to HSV-2 serostatus [15]. A study among postpartum women in Kenya similarly reported no statistically significant relationship between HSV-2 and time to CD4 count ≤200 cells/mm^3^, nor a relationship with the time to death from any cause. There was moderate risk of bias towards the null in this study because of some acyclovir use [17]. Again, differences in the outcome definitions precluded meta-analysis.

**Figure 2:**
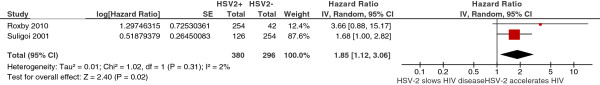
Forest plot of the impact of HSV-2 seropositivity on time to opportunistic infection/AIDS (unadjusted analysis).

Two studies considered AIDS or OI as endpoints: the Kenyan study described above [17], and an Italian study of HIV seroconverters at high risk of bias of uncertain impact [18]. Both used clinical endpoint definitions and each observed a trend towards an accelerated time to these events with HSV-2 seropositivity [17,18]. Meta-analysis was conducted to pool the univariate hazard ratios for clinical AIDS from the two studies, giving a statistically significant pooled HR=1.85 (95% CI=1.12,3.06; n=676 participants; *I*^2^=2%, Figure 2). However, this pooled analysis does not explicitly adjust for an important potential confounder, the stage of HIV disease at which individuals began observation. In the Kenyan study, an analysis adjusted only for baseline CD4 count produced an adjusted HR=1.16 (95% CI=0.52,2.56) [17], whereas the univariate hazard ratio from the Italian study, an inception cohort which inherently accounted for participants’ date of HIV seroconversion, was 1.68 (95% CI=1.19,2.37) [18]. Of note, it was not possible to conclusively exclude the possibility of some ART use in the latter study, in that it was conducted between 1983–1998 in Italy. Of further note, most (at least 44/51) of the OIs observed in the Kenyan study were conditions that can arise at any CD4 count including tuberculosis and zoster, which are arguably less indicative of HIV disease progression than other AIDS-defining illnesses (ADI) [17]; data on the types of ADI in the Italian study were not available. In conclusion, while these two studies suggest a relationship between HSV-2 seropositivity on hastening progression to first OI or clinical AIDS, other available data do not support a relationship between HSV-2 serostatus and HIV disease progression overall.

#### Plasma viral load

Five studies compared plasma HIV viral load according to HSV-2 serostatus (Table 4). Of the four reporting on prevalent HSV-2 seropositivity, one used repeated measures regression models to assess viral loads over roughly half a year [13], two reported changes in viral load at fixed time points [15,17], and one used linear regression to assess the viral load set point, defined as the numeric average of the 8- and 12-week post-infection levels [12]. None observed a statistically significant association with increased HIV viral load, including the Kenyan study described above which did observe an accelerated time to OI [17]. Similarly, no effect on change in viral load was observed in the single study examining incident HSV-2 seropositivity, although the sample size was small with only 9 seroconverters [14].

**Table 4:** **Impact of HSV-2 seropositivity on viral load**^**a**^

**Study**	**Population**	**N**	**HSV2 Ascertainment**	**Outcome: VIRAL LOAD (in log**_**10 **_**copies/mL)**				
				**Outcome**	**HSV2 pos**	**HSV2 neg**	**Difference**	**Effect**^**b**^
*Studies in acute HIV seroconverters*								
Barnabas 2011	HIV seroconverters, Americas	88	Serology: WB	VL setpoint (mean of 8 & 12 week post-infection VL)^c^	+0.3 (95% CI = −0.1,0.7)	Referent	“No significant difference”	↔
Cachay 2007	Male HIV seroconverters, USA	294	Serology: Focus + WB	VL over median of ~200 days	NR	NR	Difference “close to zero”	↔
Cachay 2008	Male HIV seroconverters, USA	9	Seroconversion to HSV2 by WB	Change in VL after vs. before HSV-2 sero-conversion over 779d	+0.17 (range −1.58, 0.49)	Reference: Before seroconversion	p = 0.57	↔
*Studies in chronic HIV infection*								
Crum-Cianflone 2006	USA	271	Serology: Focus	Change in VL at 1 year	+0.17	+0.08	+0.1, p = 0.61	↔
		221		Change in VL at 3 years	+0.46	−0.09	+0.6, p = 0.44	↑?
Roxby 2011	Postpartum women, Kenya	296	Serology: Focus	Change in VL over mean 18 months	NR	NR	No difference	↔

^a^CI = confidence interval, GUD = genital ulcer disease, MSM = men who have sex with men, OR = odds ratio, SD = standard deviation, VL = viral load, WB = Western Blot.

^b^Arrows show directions of association with HSV-2; ‘?’ denotes borderline statistical significance or clinically significant differences not meeting statistical significance.

^c^Based in part on imputed data as 47% of VL observations missing.

#### CD4 count

Three studies examining the relationship between prevalent HSV-2 seropositivity and CD4 count reported conflicting results: a marginally slower rate of CD4 count decrease among HSV-2 infected American women in a study at moderate risk of bias towards the null [12], no difference in rates of CD4 count change in a Kenyan study at moderate risk of bias towards the null [17], and larger decreases in CD4 counts after fixed time points in an American study in which several methodologic issues may have had conflicting biases (Table 5) [15]. Incident HSV-2 seropositivity was associated with no change in CD4 counts based on one study [14].

**Table 5:** **Impact of HSV-2 seropositivity on CD4 count**^**a**^

**Study**	**Population**	**N**	**HSV2 Ascertainment**	**Outcome: CD4 COUNT (in cells/mm**^**3**^**)**				
				**Outcome**	**HSV2 pos**	**HSV2 neg**	**Difference**	**Effect**^**b**^
*Studies in acute HIV seroconverters*								
Cachay 2008	Male HIV seroconverters, USA	9	HSV2 seroconversion by WB	Change in CD4 after vs before HSV-2 sero-conversion over 779 days	−44 (range = −82,220)	Reference: Before seroconversion	p = 0.36	↔
*Studies in chronic HIV infection*								
Crum-Cianflone 2006	USA	271	Serology: Focus	Change in CD4 at 1 year	−116	+14	−130, p=0.003	↓
		221		Change in CD4 at 3 years	−227	−85	−142, p = 0.03	↓
Roxby 2011	Postpartum women, Kenya	296	Serology: Focus	Rate of change in CD4 over mean 18 months	−4.22/month	−3.42/month	−0.8/month p = 0.57	↔

^a^CI = confidence interval, IQR = interquartile range, NR = not reported, MSM = men who have sex with men, WB = Western Blot.

^b^Arrows show directions of association with HSV-2; ‘?’ denotes borderline statistical significance or clinically significant differences not meeting statistical significance.

### Studies evaluating HSV-2 activity

There were no studies that met our eligibility criteria that described the relationship between HSV-2 disease activity and longitudinal measures of HIV disease progression. A single study examined the impact of HSV-2 activity on plasma HIV viral load among HSV-2 seropositive women in Burkina Faso, and observed modest increases of 0.4 log copies/mL associated with the presence of genital HSV-2 DNA over a 6-visit, 12-week follow-up period [16].

### Subgroup and sensitivity analyses

Because most results could not be pooled in meta-analyses, quantitative subgroup and sensitivity analyses were not performed, and there were not enough studies available to provide meaningful subgroup analyses based on prespecified criteria (incident HSV-2, incident HIV, geographical location). No studies examined the impact of HSV-1 co-infection. Restricting the review to studies at low risk of bias left only three studies, which collectively described a lack of association between HSV-2 seropositivity and either HIV viral load or CD4 count [13,14], but a positive association between laboratory confirmed presence of genital HSV-2 and increased plasma viral load [16].

## Discussion

This systematic review did not identify conclusive evidence of an association between HSV-2 seropositivity and HIV disease progression, changes in plasma viral load, or CD4 cell count. Although two studies observed trends towards an accelerated progression to clinical AIDS or first OI [17,18], most of the OIs observed in the study for which data were available can occur at high CD4 counts and may not represent progression of HIV [17]; further, compelling data linking HSV-2 seropositivity to other disease progression outcomes were lacking. One study found evidence that HSV-2 disease activity, defined as the presence of genital HSV-2 using PCR, is associated with increased viral load among HSV-2 seropositive individuals.

These observations must be considered in light of unequivocal data from interventional studies linking anti-HSV medications to decreased HIV viral loads and attenuated disease progression. In a meta-analysis of seven randomized trials of acyclovir or valacyclovir among HAART-untreated, HIV, HSV-2 co-infected persons, the summary treatment effect for HSV-2 suppression on plasma HIV RNA was −0.33 log copies/mL (95% CI=−0.56,-0.10) [19]. Further, this effect appears to be dose-responsive; stratification by drug demonstrated greater decreases in viral load with valacyclovir than acyclovir in the meta-analysis [19]. A meta-analysis from the pre-HAART era suggested that >3200 mg per day of acyclovir offered a significant survival benefit (hazard ratio, HR for mortality=0.78, 95% confidence interval, CI=0.65,0.93) [20], but it is unclear whether the improved survival was only related to eradication of herpesviruses (including HSV and CMV) or whether there could have been a secondary impact on HIV replication. Most recently, in the Partners in Prevention HSV/HIV Transmission Study of acyclovir 400 mg twice daily, the HR for the composite primary endpoint of CD4 count ≤200 cells/mm^3^, antiretroviral initiation, or death from non-traumatic causes, was 0.84 (95% CI=0.71,0.98) [6]. In a similar trial from the Rakai district of Uganda, the composite primary endpoint was slightly different (CD4 count ≤250 cells/mm^3^ or the occurrence of any WHO stage 4 clinical event not including esophageal candidiasis), and the HR associated with acyclovir use was 0.75 (95% CI=0.58,0.99) [5].

Indirect benefits of HSV-2 suppression are thought to primarily underlie these effects, and the observation of increased HIV viral load with HSV-2 disease activity in the Burkina Faso study supports this hypothesis [16]. But how might these findings be reconciled with the lack of convincing evidence of an effect of HSV-2 seropositivity on markers of HIV disease progression observed in this review? One explanation may be that all HSV-2 serology assays have limited sensitivity in early infection [21,22], and no studies repeated testing on seronegative participants to test for seroconversion. However, the magnitude of this effect should be minimal, given the reported incidence of HSV-2 in HIV-infected populations of 1.8 to 7.3 per 100 person-years [14,23]. A more plausible hypothesis is that considering only HSV-2 serostatus ignores the duration and activity of HSV-2 infection. Since the natural history of HSV-2 is characterized by a decreasing frequency of reactivations over time [24-27], overrepresentation of individuals with longstanding infection may produce HSV-2 seropositive cohorts with relatively inactive HSV-2. The magnitude of HIV upregulation by HSV-2 may instead depend on the degree of HSV-2 activity and be greatest during symptomatic herpes, as has been observed elsewhere [28]. Studies of HSV-2 seropositivity cannot distinguish between periods of viral latency and reactivation, such that any impact of HSV-2 activity on HIV replication may have been diluted in some of the studies we included. In support of this notion, symptomatic genital ulcer disease of any etiology has been linked to accelerated HIV disease progression [2,17,29]. It is also likely that the positive relationship between HIV viral load and HSV-2 activity is partially confounded by HIV-related immunosuppression.

In contrast to the 0.4 log copies/mL increase in viral load associated with HSV-2 activity in the Burkinabé study [16], a recent randomized trial observed larger viral load decreases of −1.23 log copies/mL using valacyclovir 1.5g twice daily [30]. Taken together, these observations suggest that reversing HSV-2 associated increases in HIV viral load may not be the only mechanism by which such drugs attenuate HIV. Direct antiretroviral effects of acyclovir and related drugs may also contribute, as have been observed in vitro by two independent groups [7,8]. Arguing against this possibility, however, an evaluation of 168 HIV infected clinical trial participants receiving either acyclovir 400 mg twice daily or valacyclovir 500 mg twice daily for 8–104 weeks failed to identify the V75I reverse transcriptase mutation that confers acyclovir resistance most commonly observed in vitro [31]. The purported explanation for this negative finding is the considerably lower concentrations of acyclovir achieved in the clinical setting compared with the laboratory studies. Still another possibility is that acyclovir could attenuate HIV disease progression by decreasing HSV-2-related immune activation, although two studies have recently shown no benefit of valacyclovir on a variety of inflammatory markers [32,33]. Further data are needed to clarify the relationship between HSV-2, anti-herpes medications and HIV viral load.

Strengths of this systematic review include our broad, multi-modality search strategy and the lack of language restrictions. There are also limitations that warrant consideration, and the robustness of our conclusions is dependent on the available evidence. First, few studies addressed our primary research question, restricting our ability to quantify the impact of HSV-2 seropositivity on HIV disease progression. Second, the measures used to define HSV-2 seropositivity and HSV-2 activity differed between studies. Although most type-specific serology assays have high, comparable levels of sensitivity and specificity when compared against the Western Blot gold standard, uncertainty regarding the optimal cutoff values in different populations and limitations of all assays in detecting early HSV-2 seroconversion may result in misclassification [34]. Third, our assessment of the risk of study bias was necessarily subjective. However, we systematically applied criteria based on the STROBE statement as has been advised elsewhere, because of the lack of a single obvious candidate tool for this purpose in systematic reviews of observational studies [11]. Fourth, differences in study populations and HIV clades may explain some observed differences between studies. Finally, inadequate control for confounding by variables such as anti-HSV drug use could have biased studies towards the null; further, no study systematically excluded HIV long-term non-progressors.

## Conclusions

This systematic review found no definitive evidence that HSV-2 seropositivity is associated with accelerated HIV disease progression in HAART-untreated persons, and modest evidence that HSV-2 disease activity is associated with increased HIV viral load. Attenuating effects of acyclovir on HIV disease progression observed in recent trials may result both from direct anti-HIV activity as well as from indirect benefits of HSV-2 suppression. Further research is needed to clarify the exact mechanisms by which HIV, HSV-2 and drugs like acyclovir interact.

## Competing interests

The authors have no competing interests to declare.

## Authors’ contributions

DT and SW conceived the study idea; DT designed the protocol, performed the analyses and wrote the original version of the manuscript; DT and KM performed the literature review and extracted relevant data; PS provided support regarding study design and analysis; all authors provided input into and approved the final version of the manuscript.

## Pre-publication history

The pre-publication history for this paper can be accessed here:

http://www.biomedcentral.com/1471-2334/13/502/prepub

## Supplementary Material

Additional file 1 “Medline search strategy”, is a Microsoft Word file (extension .docx) containing details of the strategy used for searching that electronic database. Click here for file
