# Supplementary material for: Herpes simplex virus type 2 and HIV disease progression: a systematic review of observational studies
Source: BMC Infect Dis. 2013 Oct 28;13:502. doi: 10.1186/1471-2334-13-502 (PMC3819722; doi:10.1186/1471-2334-13-502)
Supplement: Additional file 1 — “Medline search strategy”, is a Microsoft Word file (extension .docx) containing details of the strategy used for searching that electronic database. [file 1471-2334-13-502-S1.doc]

**MEDLINE search strategy**

1. simplexvirus/ or herpesvirus 1, human/ or herpesvirus 2, human/ or herpes simplex/ or herpes genitalis/ or herpes labialis/ or stomatitis, herpetic/ or ((herpes adj2 simplex) or hsv1 or hsv2 or hhv1 or hhv2 or (cold adj2 sore*) or (fever adj2 blister*)).mp.
2. exp HIV/ or exp HIV infections/
3. 2 and 1
4. exp case-control studies/ or exp cohort studies/ or cross-sectional studies/
5. exp prognosis/ or exp disease progression/ or exp morbidity/ or exp mortality/ or exp survival analysis/ or natural history.mp. or cd4 lymphocyte count/ or cd4-cd8 ratio/ or CD4-Positive T-Lymphocytes/ or CD4.mp. or exp Viral Load/
6. 4 or 5
7. 3 and 6
